# Resistance phenotypes and molecular characteristics of Staphylococcus aureus associated with pleuritis in patients at “Hôpital du Mali” teaching hospital

**DOI:** 10.21203/rs.3.rs-3579825/v1

**Published:** 2024-05-23

**Authors:** Aimé Césaire Kalambry, Tchamou Malraux Fleury Potindji, Ibrehima Guindo, Ambara Kassogue, Dinanibè Kambire, Boubacar Sidiki Ibrahim Dramé, Sadio Yéna, Seydou Doumbia, Mahamadou Diakité

**Affiliations:** Laboratory Teaching Hospital “Hôpital du Mali”; Ecole Supérieure des Techniques Biologiques et Alimentaires, Université de Lomé, Lomé, Togo; Institut National de Santé Publique; Laboratory Teaching Hospital “Hôpital du Mali”; Centre National de Recherche Scientifique et Technologique (CNRST), Institut de Recherche en Sciences de la Santé (IRSS), LR-Maladies Infectieuses et Parasitaires (LR-MIP), Ouagadougou, Burkina Faso; Laboratory Teaching Hospital “Hôpital du Mali”; Service de Chirurgie Thoracique, CHU Hôpital du Mali, Bamako, Mali; University Clinical Research Center (UCRC) Bamako, Mali; Malaria Research and Training Center (MRTC), University of Bamako, Mali

**Keywords:** Bacterial pleuritic, S. aureus, multidrug resistance, virulence, Mali

## Abstract

**Background:**

*Staphylococcus aureus (S. aureus) is* one of the pathogens strongly implicated in hospital infections. Data on the resistance and molecular characteristics of this bacterium are rare in Mali.

**Objective:**

This study aimed to evaluate the antibiotic resistance patterns, virulence factors of *S. aureus* isolates from pleural fluid infections in hospitalized patients.

**Methods:**

Pleural effusion samples were obtained by thoracentesis for bacteriological examination from October 2021 to December 2022 at the “Hôpital du Mali” teaching hospital. Comorbidities such as HIV/AIDS and diabetes were assessed. Standard microbiological procedures were used for bacterial identification. The disk diffusion method was used to identify methicillin-resistant *S. aureus*. The PCR amplification method was used to detect the following genes: *lukE/D*, *sek*, *bsa*, *sel*, and *sep*.

**Results:**

This study analyzed 6096 samples from inpatients and found a pooled frequency of bacterial pleuritis of 526 (8.6%) in thoracic surgery and pediatric wards. *S. aureus* was isolated in 52 (9.88%) cases, of which 39 (75%) isolates were MRSA. There was no significant difference between the sexes (*p = 1.00*). The median age of the patients was 30 years. All *S. aureus* isolates showed resistance to penicillin-G. The leucocidin *lukE/D* toxin was detected in 7.7% of thoracic surgery patients, but *sek*, *bsa*, *sel*, and *sep* toxins were not found.

**Conclusion:**

In this study, we found a high frequency of *S. aureus* (and MRSA) in pleurisy patients at the “Hôpital du Mali”. Only the leukocidin *lukE/D* was found. The empirical treatment protocol for pleurisy may need revision. Clindamycin, linezolid, teicoplanin, daptomycin, fosfomycin, vancomycin, moxifloxacin and fusidic acid were the most active antibiotics on our isolates in this study. Infection prevention measures, active surveillance, and effective therapeutic options are recommended.

## Background

Bacterial pleuritis is a common and widespread condition with significant mortality and morbidity [1]. Pleural infections can result from a variety of conditions and factors, such as location, demographics and comorbidities [2]. Although a positive pleural fluid culture defines infection, the microbiological pro le can be highly diverse [3]. The viridans group and pneumococci are consistently among the most common isolates; anaerobic bacteria are also found. However, *S. aureus* is the most frequently isolated organism[4, 5]. Sub-Saharan Africa remains a region with significant healthcare challenges, and the prevalence and morbidity of pleural fluid infection are of great concern. A study carried out in Chad revealed a prevalence of 13.6%. Only 4% of cases were of bacterial origin, excluding tuberculous pleuritis. With another study conducted in Niger in 2016, both authors reported that the etiology of pleuritis was dominated by tuberculosis [6, 7]. Conversely, a South African study conducted over a 5-year period reported pleuritis of bacterial origin in more than half of cases. The main pathogens isolated were *S. aureus*, *Streptococcus pneumoniae*, tubercle bacillus and *Klebsiella pneumoniae*, in that order [8]. Research into pleural infection in sub-Saharan Africa has highlighted the predominance of *S. aureus* as the most common causative organism [8]. *S. aureus* can evade the defense barriers of the host immune system by producing various enzymes and toxins. In fact, a strong correlation between toxins and disease has been reported. The main *S. aureus* toxins have been divided into three groups: 1) pore-forming toxins (hemolysin-α, hemolysin-β, leukotoxin and γ-hemolysin), 2) exfoliative toxins (ET) and 3) super antigen toxins (toxic shock syndrome toxin and staphylococcal enterotoxins) [9]. When first introduced, penicillin had a considerable impact on *S. aureus* but was gradually replaced by other beta-lactam antibiotics due to the emergence of beta-lactamase-producing strains of *S. aureus*[10]. Methicillin was introduced to treat infections caused by penicillin-resistant isolates, but the emergence of methicillin resistance in *S. aureus* (MRSA) isolates has reduced the effectiveness of antibiotics. To reduce the spread of *S. aureus* infections, knowledge of virulence factors and genetic diversity can provide useful information for tailoring infection control programs. Identifying these factors could help to improve patient outcomes and reduce the burden of pleural infections in this region. Therefore, the aim of this study was to describe the clinical and molecular characteristics of *S. aureus* isolated from patients hospitalized for pleurisy in a teaching hospital in Mali.

## Methods

### Study design and population

The present study was conducted as a cross-sectional investigation from October 2021 to December 2022 at the “Hôpital du Mali” teaching hospital in the thoracic surgery and pediatric departments among patients with pleural effusions. We obtained written consent from each participant. Review and approval of the study protocol was obtained from the ethics committee of the University of Sciences, Techniques and Technologies of Bamako, identi able through the reference number 2021/228/USTTB from 06/09/2021.

### Sample collection

Pleural effusion samples were obtained by puncture (thoracentesis) for bacteriological examination, and the samples were promptly transferred to the laboratory for analysis within an hour of collection. In addition, two comorbid conditions, namely, HIV and diabetes, were sought after. For this, blood samples were collected in dry tubes and sodium fluoride tubes. After centrifugation, serum and plasma obtained from each tube were used for the determination of HIV serology and blood sugar levels, respectively.

### Bacterial identification

Pleural effusion samples obtained previously were systematically inoculated on brain-heart infusion (BHI) and an anaerobic blood culture flask and incubated at 35°C ± 2°C for 18–24 hours. Subsequently, fresh blood agar, enriched chocolate agar, and Sabouraud agar were inoculated using the aforementioned broths. The identification of *S. aureus* was performed according to standard microbiological procedures published previously [11]. The colonies were further subjected to agglutination using the Pastorex^®^ Staph plus kit (BioMérieux, Marcy Etoile-Lyon). The identification of the isolates was later verified using the Phoenix M50 automaton (BD, Stockholm, Sweden) with the identification panel (PMIC/ID-600) specifically designed for gram-positive bacteria.

### Antimicrobial susceptibility testing

The susceptibility of *S. aureus* to antibiotics was tested with a comprehensive panel of 19 antibiotics, including beta-lactams, macrolides, lincosamide, aminoglycosides, quinolones, glycopeptides, oxazolidinones, cyclins, and diaminopyrimidine + sulfonamides. Briefly, the method involved a suspension of pure bacterial colonies in sterile normal saline to a turbidity of McFarland standard 0.5 and then uniformly spreading them on Muller Hinton agar (MHA) plates with antibiotic discs. The plates were ultimately incubated at 37°C for 24 h. The zones of inhibition were measured in millimeters and classified as either susceptible (S) or resistant (R) and interpreted according to the EUCAST recommendations [12]. Quality control was ensured using the *S. aureus* ATCC 43300 strain.

*S. aureus* exhibiting inhibition zone diameters of 19 mm or less on the 30 μg cefoxitin disk were classified as MRSA.

Of note, multidrug resistance was defined by the lack of susceptibility to at least one agent in three or more antibiotic classes as described previously [13].

### DNA extraction and molecular detection of toxin genes

DNA was extracted according to the method described previously[14]. Briefly, pure colonies were suspended in 200 μl of Tris-EDTA solution, heated at 100°C for 10 minutes, and then immediately placed at −20°C for 5 to 10 minutes. After centrifugation at 12,000 rpm for 10 minutes, the obtained supernatant was used as a DNA template. Quality control of the extraction was carried out using the Thermo Scientific NanoDrop One/One^C^ instrument. For the targeted genes:

*LukE-D*, *sek*, *sep*, *sel*, *and bsa*, the primer sequences and their respective amplicon sizes are detailed in Table 1. The amplification of the genes was achieved according to a previously described protocol using 35 thermal cycles of 95°C for denaturation, 56°C for hybridization, and 68°C for elongation [15]. The PCR products were electrophoresed on a 1.5% agarose gel with ethidium bromide at 80 milliamperes for 20 minutes and visualized using ultraviolet light using a transilluminator. A 50 bp DNA ladder was used as a size standard, which covers a size range from 50 bp to 500 bp and includes ten bands.

### Comorbidity identification

The presence/absence of HIV and diabetes was assessed using the Wondfo Rapid One Step HIV 1/2 test and the ABX Pentra 400 chemistry analyzer, respectively.

### Data collection and analysis

Data were collected through a questionnaire into the Excels sheet, and the analyses were performed using Epi info version 7.2.1.0 software. The comparison of our frequencies was conducted via Fisher’s exact test. A significance level of 0.05 or less for the p value was deemed statistically significant. The determination of the proportion of multidrug-resistant isolates was made using the Antimicrobial Resistance Data Analysis package “AMR” [16] in RStudio (R-studio Team, 2020) according to the CMI 2012 guidelines [13].

## Results

### Population features

Of 6096 patients over the study period, the pooled prevalence of bacterial pleuritis in both thoracic surgery and pediatric departments was 526 (8.6%). *Staphylococcus aureus* was isolated in 52 (9.8%) of the cases. Of those 52 patients, 28 (53.8%) were male and 24 (46.2%) were female, resulting in a male/female ratio of 0.85. Statistical analysis revealed no significant difference as per a potential relationship between gender and occurrence of the condition (*p = 0.470*). The median age (interquartile range) of the patients was 30 (30–43.8) years. Patients under the age of 4 and those between the ages of 50 and 90 accounted for 11 (21.2%) of cases.

### Comorbidities

Over the data collection period, 3 (5.8%) of the patients in the thoracic surgery department were diagnosed with diabetes, while 2 (3.8%) were HIV positive. In the pediatrics department, no cases of diabetes or HIV seropositivity were identified. It must be noted that 46 (88.5%) of the patients had taken at least one antibiotic before undergoing sampling.

### Results of antibiotic susceptibility

In both the Thoracic Surgery and Pediatrics departments, all *S. aureus* strains exhibited 100% resistance to penicillin-G. In terms of oxacillin resistance, we observed rates of 63.9% and 50% in the Thoracic Surgery and Pediatrics departments, respectively. Notably, none of the pediatric isolates showed resistance to vancomycin, daptomycin, moxifloxacin, or fosfomycin. The resistance patterns of *S. aureus* strains to various antibiotics are provided in Table 2. In the Thoracic Surgery department, MRSA was represented for 26 (72.8%) cases, while in Pediatrics, it accounted for 13 (81.2%). The most effective antibiotics were fosfomycin 2 (3.8%), fusidic acid 3 (5.8%), daptomycin 3 (5.8%), vancomycin 5 (9.6%) and teicoplanin 6 (11.5%). The K and KT phenotypes of aminoglycosides were 17 (46.2%) in Thoracic Surgery and 7 (43.8%) in Pediatrics. Regarding the antibiotic resistance patterns in MRSA, a significant association was reported for the constitutive MLS_b_ phenotype (n = 39; *p ≤ 0.000*).

Of the 52 strains of *S. aureus*, 47 (90.4%) were multidrug resistant. Moreover, according to EUCAST Expert Rules version 3.3, we found 18 (34.6%) isolates exhibiting unusual resistance phenotypes [17].

MSSA had a higher and significant percentage of susceptibility to aminoglycosides 10 (76.9%) compared to MRSA 18 (46.2%) (p = 0.05). Compared to macrolides, the constitutive MLSB phenotype was 1 (7.7%) in MSSA strains and 39 (100%) in MRSA. A significant relationship concerning MRSA was found (odds ratio: 0.0769; *p = 0.000*).

### Molecular detection of toxin genes

**Figure 1:**
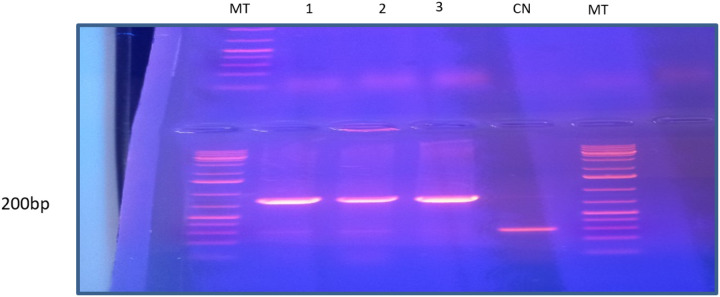
Result of lukE/D PCR MT: DNA fragment size marker (100 bp); 1; 2; 3: *S. aureus* amplicons CN: negative control; pb: base pair

In thoracic surgery, the toxin known as leucocidin *lukE/D* was detected in 7.7% of patients (n = 4), as shown in Fig. 1. The toxins known as *sek, bsa, sel*, and *sep* were not detected in any of the patients.

## Discussion

Patients referred to the Thoracic Surgery Department and the Pediatrics Department with pleurisy presented with *S. aureus* infections. The hospital frequency of pleurisy at Hôpital du Mali teaching hospital was 8.6%. Our results are lower than those reported by NGakoutou R et al, 2022, who cited in a 2006 study reporting a frequency of 15.9% in a pneumological setting. This could be explained by the evolution of practices in case management. This result is lower than those reported by some African authors (13.8% in Chad and 42.2% in South Africa) [18, 19]. In developed countries, the hospital prevalence of pleurisy was 7.75 cases in 2017 in France and 9.9 cases per 100,000 inhabitants in Finland in 2017 [20, 21]. An upward trend was found in the United States in Missouri. The weighted prevalence of empyema was 95.5 per 100,000 hospitalizations per year. It varied according to race and represented 68.10 per 100,000 inhabitants for white and black patients, respectively [22]. This increase would be linked to the emergence of bacterial strains with reduced sensitivity or expressing increased virulence. The male predominance in our study, although there was no significant difference, has been reported by other authors [23]. Most of the children in our study had their vaccination status up-to-date and had a protective effect against certain pathogens with respiratory tropism, such as *Streptococcus pneumoniae* and *Haemophilus influenzae*. These agents are indeed covered by the vaccination of the Expanded Programme on Immunization (EPI) carried out in Mali. The median age in our study was 30 years, which could be explained by the greater activity at this age of the life cycle in our country.

The issue of antibiotic resistance has been consistently identified by the World Health Organization (WHO) as a top global health priority and a critical public health menace of the current century. In Africa, the mortality rate caused by antimicrobial resistance stands out as the highest in the world, with 24 deaths per 100,000 individuals [24, 25].

In our facility, the most prescribed antibiotics to treat staphylococcal pleurisy are gentamicin (GEN) and ciprofloxacin (CIP). Our isolates showed a resistance of 38.5% to GEN and 30.8% to CIP. Our results are lower than those reported by some authors who found 72.6% resistance to GEN and 71.7% resistance to CIP [26]. For aminoglycosides, there was no significant difference between the presence of the sensitive phenotype in MRSA and MSSA (*p = 0.05*). According to the guidelines of the American Association of Thoracic Surgery (AATS) and the British Thoracic Society (BTS), aminoglycosides should be avoided in the management of pleurisy, while other authors advise (13) molecules in the treatments for MRSA infections [27, 28].

*S. aureus* penicillinase is species specific, plasmid-borne, and transmissible. It gives it resistance to penicillins G, V and A, carboxypenicillins and ureido-penicillins. More than 90% of *S. aureus* isolated from pathological products in the hospital environment are penicillinase producers [29]. In our study, penicillinase G was observed in 100% of our isolates. This observation con rms the strong trend of resistance of staphylococci to penicillin. In addition to their intrinsic resistance to beta-lactams, hospital-associated MRSA strains often show a variable but alarming level of multidrug resistance, which limits the possibilities of treatment to the few remaining effective drugs.

MRSA poses a significant threat to public health, particularly in developing countries, due to its ability to cause life-threatening infections [11]. MRSA prevalence data in Africa are variable in terms of coverage and quality. They are available for South Africa, Nigeria and the countries of the Mediterranean basin but are fragmentary for the other countries [28]. Some come from single center studies, and others come from larger but few surveillance systems. Many studies have relied on phenotypic methods to identify MRSA.

In our study, 75% of MRSA isolates were encountered. Some authors have reported an MRSA prevalence of 80.9% in Cyprus in 2022 [11]. In 2018, a study bringing together data from a large number of countries estimated the prevalence of MRSA to be between 25 and 50% for Algeria, Morocco and Cameroon compared to 10 to 25% for the Republic of Ivory Coast and Senegal; Mali had not provided data [28]. The variation between countries could be partly explained by differences in the availability and use of antimicrobials, the incidence of HIV infection (a risk factor for MRSA colonization) and differences in local infection control practices and specific pathogen characteristics of circulating clones. Due to differences in the extent of collection and testing methods, care should be taken when comparing data between countries.

For macrolide resistance, the inducible MLS _b phenotype_ was more frequent in MSSA than in MRSA (15.4%) (p = 0.04). On the other hand, the constitutive MLS _b phenotype_ was more frequent in MRSA than in MSSA (100%) (p = 0.000). We noted a 56.2% predominance of the constitutive MLS _b phenotype_ against 22.9% of the inducible MLS _b phenotype_ in Iran [30]. Compared to vancomycin, the resistance is 9.6%. No resistance to vancomycin was detected in Zambia by the authors in hospital settings [31]. Vancomycin is the drug of choice used in the hospital setting to treat MRSA infections. Our results could perhaps be explained by the possible acquisition of resistance to vancomycin. The choice of this antibiotic to treat MRSA may also cause the emergence of vancomycin resistance.

*S. aureus* is a pathogen whose strong adaptive power allows survival through the successive acquisition of antibiotic resistance genes, growth regulatory mechanisms in the presence of antibiotics and specific virulence factors. The vigilance of clinicians and microbiologists is needed to signal the emergence of new epidemiological phenomena, as well as to ensure compliance with prevention instructions and the judicious use of antibiotics, both in hospitals and in the community.

*S. aureus* has a vast arsenal of virulence factors (including adhesive, immunomodulatory, and host cell-damaging molecules) whose presence or specificity varies from clone to clone, a variability that is reflected in the wide variety of infections this bacterium can cause [11]. Many virulence genes are found on mobile genetic elements; thus, their combination differs significantly between clones and even between closely related strains. The potential association of specific virulence factors with certain types of *S. aureus infections* or their severity remains di cult to establish, probably due to the number of these factors and their redundant functions.

Certain staphylococcal toxins are responsible for specific clinical syndromes with sometimes very poor prognosis. This is Panton-Valentine Leucocidin (PVL). Leukocidin E (*LukE/D*) is a porogenic toxin produced by *S. aureus* that lyses host cells and promotes the virulence of the bacterium; it is hemolytic, unlike PVL [32].

We found 7.7% positivity for *lukE/D*. This rate is lower than those obtained by some authors, 44.3% in 2022 in China and 68.3% in 2017 in the Democratic Republic of Congo [33]. The absence of virulence factors *sek*, *bsa*, *sep* and *sel* during our study could indicate that the isolates obtained do not possess enterotoxin genes.

Clindamycin, linezolid, teicoplanin, daptomycin, fosfomycin, vancomycin, moxifloxacin and fusidic acid were the most active antibiotics on our isolates during this study. On the other hand, at the Point G teaching hospital, Maiga A et al., 2017, found that the most active antibiotics were fusidic acid, the association amoxicillin + clavulanic acid and pristinamycin [34]. In this group of antibiotics, the resistance to fusidic acid was 5.8%, which implies that this antibiotic still remains active against *S. aureus* isolates.

### Limitations

Our study was limited to a single hospital and is therefore not representative of the whole country; however, the pro le of the affected population and their behavior are typical of the country. We did not perform molecular typing (clonal complex, sequence typing, *SCCmec* typing), molecular detection of the *mecA gene* coding for methicillin resistance, and even less molecular confirmation of the phenotypic identification of *S. aureus* by detection of the *nuc* gene coding for thermonuclease.

## Conclusion

The results of our study indicate that *S. aureus* is frequent among patients with pleurisy admitted to the Hospital of Mali Teaching Hospital, and the frequency of MRSA is alarming at 75%, particularly in the absence of a national prevalence assessment. This study can serve as a reference for monitoring the development of MRSA and setting up a surveillance system at the hospital. Our findings suggest that the current empirical treatment protocol for pleurisy, which consists of ciprofloxacin and gentamicin, may need to be revised.

Despite the limited exploration of the frequency and severity of MRSA pleurisy in our country, our study highlights the importance of implementing infection prevention measures, such as the establishment of an infection control team and admission screening for MRSA. Furthermore, a better understanding of the epidemiology of these infections and the development of effective therapeutic strategies are essential for clinicians to prevent the spread of these infections.

## Figures and Tables

**Table 1:** different sequences of primers of toxin genes and references

Primer name	Primer sequence (5′ → 3′)	Size of products (pb)	References
*bsa_Forward*	ACAGAAGCTGTTAAAACTACCC	100	[12]
*bsa_Reverse*	GATTAATATGACAATTGAAGTGGGTC	100	[12]
*lukE/D_Forward*	GCAACTTTTGTCAGTAGGACTG	200	[12]
*lukE/D_Reverse*	GTCTACTTCACTGACATAACTC	200	[12]
*sek_Forward*	GGTGTCTCTAATAGTGCCAG	200	[12]
*sek_Reverse*	TCGTTAGTAGCTGTGACTCC	200	[12]
*sel_ Forward*	ATCAATGGCAAGCATCAAACAG	200	[12]
*sel _Reverse*	TGGAAGACCGTATCCTGTG	200	[12]
*sep_Forward*	GACCTTGGTTCAAAAGACACC	200	[12]
*sep_Reverse*	TGTCTTGACTGAAGGTCTAGC	200	[12]

**Table 2:** Antibiotic resistance of S. aureus isolates

	Thoracic surgery	Pediatrics	Total
Antibiotics	Resistant n(%)	Resistant n(%)	Resistant n(%)
Penicillin-G	36(100)	16(100)	52(100)
Oxacillin	29(80.6)	13(81.3)	42(80,8)
Cefoxitin	26(72,8)	13(81,2)	39(75)
Amikacin	17(47,2)	7(43,8)	24(46,2)
Gentamicin	15(41,7)	5(31,2)	20(38,5)
Tobramycin	19(52,8)	7(43,8)	24(46,2)
Levofloxacin	12(33,3)	4(25,0)	16(30,8)
Ciprofloxacin	12(33,3)	4(25,0)	16(30,8)
Moxifloxacin	9(25,0)	0(0)	9(17,3)
Tetracyclin	35(97,2)	15(93,8)	50(96,2)
Erythromycin	28(77,8)	14(87,5)	42(80,8
Clindamycin	4(11,1)	1(6,2)	40(76,9)
Linolezid	9(25,0)	4(25,0)	13(25,0)
Teicoplanin	5(13,9)	1(6,2)	6(11,5)
Daptomycin	3(8,3)	0(0)	3(5,8)
Trimethoprim + sulfamethoxazole	26(72,2)	11(68,8)	37(71,2)
Fosfomycine	2(5.6)	0(0)	2(3.8)
Vancomycin	5(13.9)	0(0)	5(9.6)
Fusidic acid	1(2.8)	1(6.2)	3(5.8)

## Data Availability

All the information supporting our conclusions and appropriate references are included in the manuscript. The datasets used and analyzed in the current study are also available from the corresponding author.

## Abbreviations

PCR: Polymerase Chain Reaction
S.aureus: Staphylococcus aureus
MRSA: Methicillin-Resistant *Staphylococcus aureus*
USTTB: University of Sciences, Techniques and Technologies of Bamako
EUCAST: European Committee on Antimicrobial Susceptibility Testing
MSSA: Methicillin-Sensible *Staphylococcus aureus*
MHA: Muller Hinton Agar
HIV/AIDS: Human Immunodeficiency Virus/ Acquired Immune De ciency Syndrome
DNA: Deoxyribonucleic Acid
EPI: Expanded Programme on Immunization
